# Acute Ozone-Induced Differential Gene Expression Profiles in Rat Lung

**DOI:** 10.1289/ehp.7413

**Published:** 2005-06-23

**Authors:** Srikanth S. Nadadur, Daniel L. Costa, Ralph Slade, Robert Silbjoris, Gary E. Hatch

**Affiliations:** 1 Pulmonary Toxicology Branch, Experimental Toxicology Division, and; 2 Clinical Research Branch, Human Studies Division, National Health Environmental Effects Research Laboratory, Office of Research and Development, U.S. Environmental Protection Agency, Research Triangle Park, North Carolina, USA

**Keywords:** acute exposure, gene expression profiles, lung, microarray, ozone, rat

## Abstract

Ozone (O_3_) is an oxidant gas that can directly induce lung injury. Knowledge of the initial molecular events of the acute O_3_ response would be useful in developing biomarkers of exposure or response. Toward this goal, we exposed rats to toxic concentrations of O_3_ (2 and 5 ppm) for 2 hr and the molecular changes were assessed in lung tissue 2 hr postexposure using a rat cDNA expression array containing 588 characterized genes. Gene array analysis indicated differential expression in almost equal numbers of genes for the two exposure groups: 62 at 2 ppm and 57 at 5 ppm. Most of these genes were common to both exposure groups, suggesting common roles in the initial toxicity response. However, we also identified the induction of nine genes specific to 2-ppm (thyroid hormone-β receptor c-erb-A-β and glutathione reductase) or 5-ppm exposure groups (*c-jun*, induced nitric oxide synthase, macrophage inflammatory protein-2, and heat shock protein 27). Injury markers in bronchoalveolar lavage fluid (BALF) were used to assess immediate toxicity and inflammation in rats similarly exposed. At 2 ppm, injury was marked by significant increases in BALF total protein, *N*-acetylglucosaminidase, and lavageable ciliated cells. Because infiltration of neutrophils was observed only at the higher 5 ppm concentration, the distinctive genes suggested a potential amplification role for inflammation in the gene profile. Although the specific gene interactions remain unclear, this is the first report indicating a dose-dependent direct and immediate induction of gene expression that may be separate from those genes involved in inflammation after acute O_3_ exposure.

The photochemical oxidant ozone (O_3_) is the air pollutant in smog thought to be of greatest concern with regard to acute health effects [U.S. Environmental Protection Agency (EPA) 1996]. Although considerable progress has been made in improving U.S. air quality since air pollution standards were established in 1970, about 50% of the U.S. population currently lives where O_3_ levels exceed the National Ambient Air Quality Standard (NAAQS) (U.S. EPA 1993). Of the six NAAQS pollutants, O_3_ has been the most problematic pollutant to control because it is formed from intermediates originating from many different sources. Hence, concerns about adverse health impacts remain. It is known that acute exposure to this gas at ambient levels results in acute lung injury and inflammation in humans (Devlin et al. 1991). Airway epithelial cells are damaged and lung function is impaired in both humans and laboratory animals (Hatch et al. 1994; Koren et al. 1989). Additionally, because O_3_ reaches the deep lung and damages distal airway and proximal alveolar structures (including the surface epithelia and connective tissues), there is a potential for permanent damage with repeated exposure and injury to the deep lung (Costa et al. 1985). Recent epidemiological studies have associated increased morbidity, particularly in children with asthma, during periods of high O_3_ pollution (Tolbert et al. 2000; White et al. 1994).

O_3_ appears to induce initial damage to the respiratory epithelium because of an oxidative cascade after its initial reaction with lipids and proteins at the air–liquid interface (Pryor 1992). Injury to the epithelium results in sloughing of ciliated cells into bronchoalveolar lavage fluid (BALF). Increased protein concentration and *N*-acetylglucosaminidase (NAG) activity in the BALF also occur because of leakage of proteins from blood plasma or intracellular spaces (Dye et al. 1999; Hu et al. 1982; Vincent et al. 1996). The release of inflammatory cytokines and chemokines from injured cells initiates the infiltration of neutrophils, which are also increased in the BALF (Devlin et al. 1991) and at least in the short run are thought to contribute to injury. Despite the evidence that this overt process wanes when repeated over time, it appears that the injury and inflammation cascade promotes cellular hypertrophy and the deposition of interstitial matrix materials and generalized remodeling of the fine structures of the deep lung (Chang et al. 1992; U.S. EPA 1993).

O_3_ is also hypothesized to initiate intra-cellular oxidative stress through ozonide and hydroperoxide formation (Pryor 1992). These intracellular oxidants are likely to activate gene transcription through redox-mediated signaling pathways that govern the cascade of injury, repair, and other cellular responses associated with the oxidant burden. For example, the inflammatory cytokines and chemokines inter-leukin (IL-8), macrophage inflammatory protein-2 (MIP-2), and cytokine-induced neutrophil chemoattractant (CINC), which are found in the BALF and lung tissues of rodents exposed to O_3_ (Michelec et al. 2002; Zhao et al. 1998), can initiate differential transcriptional activation of genes. Because gene expression is mediated by various transcription factors, which can ultimately determine the outcomes of the challenge, we hypothesized that gene expression profiles derived using gene arrays could aid in identifying exposure-specific gene regulation for O_3_ that might then lead to the identification of potential gene markers for acute lung injury. Although the inflammatory response to O_3_ has been well documented, the earliest signaling pathways associated with this process are not known.

The acute O_3_ lung injury model has been widely used to explore injury and repair processes (Bassett et al. 1988; Kleeberger et al. 1997; Prows et al. 1999). It provides a well-documented and reproducible tool to study the fundamental events associated with acute lung injury induced by oxidant overload. It was felt that oxidant-based profiles arising from this study might aid in our understanding of various biochemical pathways involved in lung injury, inflammation, and repair processes. It may also be possible to identify acute markers associated with long-term outcomes that serve to guide hypotheses generation to explore further understanding of acute lung injury.

Commercially available microarray technologies can facilitate efforts at global gene expression profiling. However, the rat genome is not yet completely sequenced, and the global approach with microarrays containing numerous expressed sequence tags may not be able to provide the needed information on possible candidate genes that can be further explored at this time. We therefore used the nylon micro-array with a limited and targeted number of well-characterized rat genes to identify gene expression profiles involved in the acute response to toxic doses of O_3_.

## Materials and Methods

### Animals.

Fischer 344 rats (male, 90 days of age) were obtained from Charles River Laboratories (Raleigh, NC) and kept in temperature- and humidity-controlled rooms with a 12/12-hr light/dark cycle. Standard rat chow (ProLab, Brentwood, MO) and water were provided ad libitum. The animal facility is Association for Accreditation of Laboratory Animal Care approved, and all procedures were reviewed and implemented through the Institutional Animal Care and Use Committee process of the U.S. EPA National Health and Environmental Effects Research Laboratory.

### Inhalation exposures.

Rats (six animals per group) were placed in individual stainless-steel wire-mesh cages inside a 135-L exposure chamber and exposed to either 2.0-ppm O_3_ or 5.0-ppm O_3_ for 2 hr. Control animals were exposed to filtered room air. Chamber O_3_ concentration was monitored with a Dasibi model 1003AH O_3_ monitor (Dasibi Environmental Corp., Glendale, CA).

### Lung removal.

Two hours postexposure, rats were anesthetized by an ip injection of (50 mg/kg body weight) pentobarbital (Abbott Laboratories, North Chicago, IL) and exsanguinated by severing the dorsal aorta. The chest cavity was opened, and the lungs were removed en bloc. Individual lobes were separated, quick frozen in liquid nitrogen, and stored at −80°C until used for RNA extraction.

### Bronchoalveolar lavage.

Rats exposed identically to those used for gene expression analysis were also anesthetized and bled. A tracheal cannula was inserted to about 0.5 cm above the carina, and the whole lung was lavaged three times with the same volume of isotonic 0.85% NaCl (Ca^2+^ and Mg^2+^ free) that had been warmed to 38°C. A volume equal to 30 mL/kg of body weight was injected and reinjected 3 times in succession. This saline was then withdrawn and placed on ice. Cells were separated by centrifugation at 1,100 × *g* for 15 min at 4°C. Aliquots of the supernatant were taken for protein and enzyme assays. The cell pellet was resuspended in saline and separated into two fractions. One fraction was stained with 0.6% crystal violet in 4% acetic acid and counted in a hemocytometer to obtain the total cell count. The other fraction was cytocentrifuged (Shandon, Inc., Pittsburgh, PA) onto a microscope slide and stained for differential cell counting using Diff-Quik stain (Fisher Scientific, Pittsburgh, PA). Total protein in the bronchoalveolar lavage (BAL) supernatant was assayed using the method of Bradford (1976), with bovine serum albumin as standard. NAG was measured from the hydrolysis of *p*-nitrophenyl-*N*-acetyl-β-d-glucosamine, using *p*-nitrophenol as standard (Vincent et al. 1996). Lysozyme was measured by the *Micrococcus* lysis method (Konstan et al. 1982).

### RNA extraction.

Rats exposed exclusively for the gene expression studies did not undergo BAL to avoid confounding of the gene expression that might be associated with the physical stress of lavage or the loss of desquamated cells. Total RNA was extracted from lung lobes dissected free of the trachea, using Trizol reagent (Invitrogen, Carlsbad, CA). RNA was treated with DNAse (Invitrogen) to remove any contaminating DNA and purified after phenol:chloroform extraction. Quantity and quality of RNA was checked by ultraviolet spectrophotometer and formaldehyde gel analysis (Sambrook and Russell 2001). To ensure adequate RNA sample size and to minimize variability between samples in this exploratory study, we implemented a system of sample pooling. From the six rats of each exposure group, three pooled samples of two rats were created randomly. A fourth sample was generated by pooling RNA from all six animals at a ratio equal to a normalized group sample. This method was modified from similar pooling procedures followed in gene array studies (Liu et al. 2003; Noh et al. 2004).

### Atlas cDNA array analysis.

Rat cDNA expression array containing 588 cDNAs (spotted in duplicate) on a nylon membrane was purchased from Clontech (Palo Alto, CA) and used in this study. GenBank accession numbers for these genes provided by Clontech were derived from the National Center for Biotechnology Information (NCBI) UniGene database (http://www.ncbi.nlm.nih.gov). Total RNA (15 μg) was converted to ^32^P-labeled cDNA in a reverse transcriptase reaction following the manufacturer-suggested protocol, with a slight modification. The reaction was extended for 15 min after the addition of cold 40 μM dATP to improve the quality of the probe (Nadadur and Kodavanti 2002). ^32^P-labeled cDNA probes were separated from unincorporated nucleotides using a spin column (Nucleospin extraction kit, Clontech), and the efficiency of ^32^P incorporated into cDNA was measured by scintillation counting. The rat Atlas cDNA array was hybridized with ^32^P-labeled cDNA probes overnight at 60°C. The microarrays were washed to highest stringency condition (two 20-min washes in 0.1 × saline–sodium citrate and 0.1% sodium docecyl sulfate). The nylon membranes were exposed to a phosphor screen for 4 hr, and array blot images were scanned using a Phosphorimager (Molecular Dynamics, Piscataway, NJ). Four array hybridizations were performed for each group.

### Microarray data analysis: quality control and quality assurance measures.

The scanned images were aligned using AtlasImage software (version 2.7; Clontech). The spot intensities (gene expression) were globally normalized and corrected for background with the median setting following the protocols defined in the AtlasImage software, version 2.7. Spot density values for all the genes were imported to GeneSpring software (version 6.0; Silicon Genetics, Redwood City, CA) and subjected to quality control (QC) measures to identify the total number of genes that showed hybridization signals above the background in all 12 arrays (four arrays per group). The QC gene list generated was analyzed to identify altered genes using a filter of 2-fold change.

### Statistical analysis.

Gene lists generated (for genes either induced or suppressed by 2-fold) were subjected to statistical analysis using the GeneSpring preprogrammed statistical package. Genes whose expressions were altered by 2-fold were subjected to one-way analysis of variance (ANOVA) setting *p*-values of < 0.05. The comparison is performed for each gene in all the groups, and the genes with the set cutoff (*p*-values of < 0.05) are returned. The genes selected by one-way ANOVA were also corrected for false rate discovery following the Benjamini and Hochberg (1995) method. Gene lists (induced/suppressed) generated in this way were used in Venn diagram analysis to identify the genes that were common or unique to each exposure group (2 or 5 ppm) and were listed.

### Real-time reverse transcriptase PCR.

Relative gene expression was quantified using real-time reverse transcriptase (RT) quantitative PCR on selected genes to verify the microarray data. Total RNA (5 μg) was reverse transcribed to generate first-strand cDNA using Moloney murine leukemia virus reverse transcriptase (Invitrogen) and random primer mix (Invitrogen). Taqman predeveloped assay reagents (Applied Biosystems, Foster City, CA) were used for amplification of induced nitric oxide synthase (*Nos2*), *Jun*, and glyceraldehyde-3-phosphate dehydroge-nase (*GAPDH*). Oligonucleotide primer pairs for thyroid hormone-β receptor (*Thrb*) glutathione reductase (*Gsr*) were designed using a primer design program (Primer Express, Applied Biosystems) and obtained from Integrated DNA Technologies (Coralville, IA). Quantitative fluorogenic amplification of cDNA was performed using the ABI Prism 7700 Sequence Detection System (Applied Biosystems). The relative abundance of mRNA levels was determined from standard curves generated from a serially diluted standard pool of cDNA prepared from human bronchial epithelial cells. The relative abundance of *GAPDH* mRNA was used to normalize levels of the mRNAs of interest.

## Results

### Bronchoalveolar lavage fluid analysis.

The indicators for lung injury and inflammation measured in BALF 2 hr after the 2-hr exposure to air or 2 or 5 ppm O_3_ are presented in Table 1. BALF protein concentrations were increased significantly by 20-fold in the 5-ppm group but were changed only about 1.5-fold in the 2-ppm group. NAG was increased 7.5-fold in the 5-ppm group and 1.5-fold in the 2-ppm group. Lysozyme was not significantly affected in either exposure group. Total cell counts appeared to be decreased by about 20% after both the 2- and 5-ppm exposures. This decrease is common to O_3_-exposed animals immediately after exposure because it is thought that macrophages become activated and are not available to BAL. Neutrophil and ciliated cell percentages in the BALF (which are normally close to zero) increased significantly in both the 2- and 5-ppm groups in a concentration-dependent manner. However, this increment at 2 ppm, although significant, was in the range of BALF neutrophils considered “normal” for control rats. Had BAL been conducted 12–15 hr postexposure, as is more typical (Hatch et al. 1986), it is likely that these values would have been considerably higher. Notably, however, in the 5-ppm group, the neutrophils and ciliated cells were substantially increased to 23 and 40%, respectively, of total cells, indicative of concomitant immediate airway and alveolar damage and inflammation.

### Microarray analysis.

Analysis of the expression of 588 genes spotted on the rat cDNA nylon array showed that 540 genes were expressed constitutively in the lung of all the treatment groups including controls. With exposure to O_3_, statistically significant augmentation (with 2-fold set as a minimal induction threshold in the statistical analysis) of expression was found in 62 genes at 2 ppm and 57 genes at 5 ppm O_3_. Of these genes, 26 were induced commonly in both 2- and 5-ppm exposure groups, and a total of 36 genes in the 2-ppm group and 31 genes in the 5-ppm group were suppressed (Table 2). Despite the difference in the exposure concentration, the immediate toxic response appeared to be mediated by the transcriptional regulation of many common genes: induction of 17 and suppression of 25 genes in both exposure groups. Further analysis indicated concentration-specific induction and/or suppression of unique genes (Table 2), suggesting their possible roles in initiating different downstream signaling networks. The up-regulated genes that were common to both 2 and 5 ppm O_3_ treatment are listed in Table 3; the common down-regulated genes are listed in Table 4. Induced genes unique to both the 2- and 5-ppm exposure groups are listed in Table 5. Similarly, suppressed genes that are unique to the 2- and 5-ppm exposure groups are listed in Table 6.

Of 13 functional groups represented on this microarray, O_3_-altered gene expression profiles were distributed predominantly into four broad functional groups: *a*) metabolism (lipid, protein), *b*) intracellular transducers/stress response (modulators, oncogenes), *c*) growth factors/receptors (kinases, activators/inhibitors), and *d*) cell surface receptors (adhesion proteins and ligands). Among these groups, stress-response proteins, oncogenes, and cell cycle–related genes were up-regulated, whereas cell surface receptors were down-regulated. Lipid metabolism genes were differentially expressed in response to O_3_ inhalation. The altered expression in lipid metabolism and the transcription factors nuclear factor κB (*Nfkb1*), *ras* oncogenes, and insulin-like growth factor (IGF) binding protein-2 (*Igfbp2*) and the concentration-specific differential expression of stress-response proteins such as *Jun*, *Gsr*, and calcium-dependent signal mediators, observed in the present study for the first time, will shed new light on their possible roles in acute O_3_ toxicity. Further analysis of the altered expression of genes unique to 2 or 5 ppm (Tables 5, 6) will be more useful in identifying exposure-specific immediate lung injury.

To validate the altered gene expressions observed in the microarray assessment, real-time RT-PCR was performed on five selected genes (four of which were not known to be associated with O_3_ toxicity, and one known gene was found altered in rat lung tissue on exposure to O_3_). As shown in Table 7, the expression of these five genes is in good agreement with the microarray analysis.

## Discussion

The studies we report here represent part of our ongoing effort to characterize the immediate biologic responses of rat lung tissue to a toxic dose of O_3_ and to use this information to develop biomarkers for its toxicity (Hatch et al. 1986, 1994). This effort was to generate gene expression profiles for rat lung tissue using high-throughput microarray technologies to distinguish levels of injury based on the differential expression of specific groups of genes thought to be involved in this process. The gene expression profiles derived at 2 hr after O_3_ inhalation represent toxicant-induced transcriptional activation/inactivation that is not likely confounded by other physiologic factors as might occur after established inflammation. To the best of our knowledge, our present study is the first to be published on the near-immediate impact of acute O_3_ exposure on gene expression response profiles in rat lung tissue. Two related reports on O_3_-altered gene expression profiles have appeared in the literature. One involved mice (Gohil et al. 2003) assayed after repeated O_3_ exposures (1 ppm; 8 hr/day) for 3 days, with analysis performed immediately after the third exposure. Another investigation was carried out in rats exposed to 1 ppm O_3_ for 3 hr (Bhalla et al. 2002) and evaluated for the expression of inflammatory marker genes at a relatively late time point (10–12 hr postexposure). In both studies it is likely that significant inflammation and repair processes were involved. In contrast, gene expression profiles derived in the present study represent the near-immediate transcriptional alterations in response to a single exposure to a toxic dose of O_3_ and, not surprisingly, present a profile different from these other studies.

In the present study we exposed rats to 2 and 5 ppm of O_3_ for 2 hr. The 2-ppm exposure was selected to represent a possible human exposure during vigorous human exercise at a high exposure concentration of approximately 0.4 ppm of O_3_ (Hatch et al. 1994), whereas the higher level (5 ppm) might represent a more severe oxidant challenge that may initiate acute respiratory distress syndrome involving concomitant oxidant injury and inflammation. Using ^18^O-labeled O_3_, we (Hatch et al. 1994) have shown that the impact of acute exposure to O_3_ at 0.4 ppm with intermittent heavy exercise in humans resulted in lung tissue dosimetry approximately equal to that of the rat exposed sedentary to 2 ppm for the same 2-hr period.

The initial interaction of O_3_ with the unsaturated fatty acids in the epithelial lining fluid is thought to generate lipid ozonation products that drive various signaling cascades that result in the biochemical events characteristic of O_3_ pulmonary toxicity. As such, the immediate molecular changes leading to gene induction at this step may be identifiable using high-throughput technologies leading to candidate biomarkers for O_3_ exposure and toxicity. Thus, induced genes may ultimately lead to the development of markers that can be screened using noninvasive approaches (Krishna et al. 1998; Liu et al. 1999).

The airway epithelium is the first line of defense against inhaled toxicants and also is the primary site of O_3_-induced injury (Koren et al. 1991). Acute exposure to O_3_ leads to immediate epithelial injury, pulmonary neutrophilic inflammation subsequent to permeability changes, and the leakage of serum proteins into the air spaces of the lung. The increase in BALF protein content, NAG activity, and recoverable neutrophils are collectively indicative of airway and alveolar epithelial necrosis. This pattern of markers and inflammatory cellular response is typically observed at later time points (12–18-hr postexposure) as markers of exposure and injury (Bhalla and Gupta 2000; Hatch et al. 1994; van Bree et al. 2001). The earliest cellular and molecular events are generally not studied because of lack of sensitive tools.

The statistically significant differences in the expression of 119 genes in the two exposure groups together suggest that immediate transcriptional regulation of these genes may be involved in the tissue injury and/or regenerative responses. The gene expression data derived in the present study suggest that the O_3_-induced injury is mediated by differential activation of genes predominantly distributed in two groups: fatty acid metabolism and cell proliferation. In contrast, genes representing signal mediators, receptors, or second messengers were suppressed. Interestingly, the altered gene expression profiles of the two exposure groups (2 and 5 ppm) indicated that most genes affected were common (Tables 3, 4). It remains to be seen if the response generalizes to other oxidants.

The 3.5-fold induction in the expression of the adhesion molecule L-selectin observed 2 hr after exposure to 2 and 5 ppm O_3_ suggests its role in the migration and increased accumulation of neutrophils observed at this early time point. Induction of other adhesion molecules, including P-selectin, has been observed in human BALF cells on acute exposure to 0.12 ppm of O_3_ (Blomberg et al. 1999; Krishna and Holgate 1999). Increased expression of apurinic and apyrimidinic (AP) endonuclease (~ 5-fold) indicates possible activation of DNA repair processes (He et al. 2001). Simultaneous induction of β-arrestin-1 and β-arrestin-2, along with cyclins, clearly suggests the initiation of epithelial cell DNA repair and subsequent cell proliferation. Besides, β-arrestin proteins, which belong to the G-protein–coupled receptor family, are also known to act as scaffold proteins that mediate the activation of MAP kinase cascades (Luterrel et al. 2001; Sun et al. 2002).

The differential activation of lipid metabolism genes (induction of fatty acid amide hydrolase, phopholipase A2–activating protein) agrees with the long-known biochemical evidence of lipid ozonation products generated from the phospholipid pools of the pulmonary surfactant or the epithelial cell membranes (Kafoury et al. 1999). *In vitro* O_3_ exposure also has been shown to activate phospholipase A2, C, and D in cultured epithelial cells (Wright et al. 1994). The consequences of altered expression of phospholipases and the generation of lipid signal transduction network elements in response to lipid ozonation products are complex (Kafoury et al. 1999). Lipid signal transduction networks involve cross-talk among various isoforms (Liscovitch 1992). The altered expression of genes involved in lipid metabolism suggests their possible involvement in initiating a cascade of biochemical events that can lead to cellular responses characteristic of O_3_ toxicity in the lung.

The present study also indicated dose-specific unique gene expression profiles. The high dose of 5 ppm induced the expression of various stress-response genes such as the transcription factor *Jun*, *Nos2*, *MIP-2* (*Cxcl2*), and heat shock protein 27 (*Hspb1*). This is the first observation of such an immediate induction of these genes. Although the induced expression of heat-shock proteins MIP-2 and Nos2 has been reported at later time points such as 4–8 hr after exposure to 2 ppm O_3_ (Driscoll et al. 1993; Johnston et al. 2001; Zhao et al. 1998), the induction observed here occurred within 2 hr after 2 hr of 5 ppm but not 2 ppm. The induction of MIP-2 and Nos2 only in the rat lungs exposed to 5 ppm O_3_ suggests their participation in or the result of the rapid and immediate influx of neutrophils observed in this group. Induction of *Jun* and *Hspb1* in rat lungs exposed to 5 ppm O_3_ suggests a role in downstream signaling of stress-response cascade(s). Understanding the relationships and roles of these genes provides novel insight as to the mechanisms of oxidant toxicity and subsequent adaptive responses. Conversely, *Thrb* and *Gsr* were induced exclusively in 2-ppm–exposed animals compared with 5 ppm, suggesting a toxic response specific to the lower dose of O_3_.

The role of hormonal factors, particularly thyroid hormone, in O_3_ toxicity has been recognized previously (Fairchild and Graham 1963). Recent studies by Huffman et al. (2001) showed that a 2-fold increase in circulating thyroid hormone levels appeared to enhance pulmonary toxicity to short-term inhalation to 2 ppm O_3_ in rats, suggesting a role for this hormonal reflex. Thyroid hormone has been shown to regulate its own receptor, and the protooncogene *c-erbA* has also been identified as a thyroid hormone receptor. Three of the four *c-erbA* gene products—erbA-α1, erbA-β1, and erbA-β2—encode biologically active thyroid hormone receptors (Teboul and Torresani 1993). Hyperthyroidism in rats produces organ hypertrophy and an increase in circulating levels of IGF and its binding proteins (IGFBP) (Rosato et al. 2002). IGF-1 is the major mediator of growth hormone effects (Iglesias et al. 2001). It has also been observed that expression of IGF and IGFBP may mediate the number and density of thyroid hormone receptors (Pellizas et al. 1998). The 5-fold induction in the expression of thyroid hormone receptor *Thrb* and 5- to 15-fold suppression in IGF-binding protein are the first observations of O_3_-induced alterations in thyroid hormone receptor expression and regulation of *Igfbp2*. These observations suggest the possible role of *Thrb* and *Igfbp2* in the increased O_3_ toxicity observed in hyperthyroid rats (Huffman et al. 2001).

Immediately altered gene expression pro-files derived for the rat lung upon exposure to toxic doses of O_3_ indicated altered expression of an array of genes common to both the concentrations studied (2 and 5 ppm), whereas some were unique to each dose. These gene profiles represent a spectrum of initiating events and recovery responses. The induced genes involved fatty acid metabolism, cell proliferation, and stress response, and the suppressed genes involved signal mediators, second messenger systems, and G-protein–coupled receptors. The observation of differential expression of *Igfbp2* and *Thrb* provides the first biochemical clue for their involvement in O_3_ toxicity and its exacerbation in hyperthyroid conditions. Increased expression of genes involved in cell proliferation, DNA damage repair, and the stress response, such as *Nos2*, *Gsr*, and transcription factors c-jun and NF-κb, suggests the initiation of injury recovery response pathways. Further detailed analysis of these genes and their downstream signaling pathways may shed light on their roles, and they may serve as potential biomarkers for monitoring O_3_ toxicity. The gene expression profiles presented here were derived from total lung tissue, which could have in part masked or diluted the injury response in airway epithelium. Alternatively, marginated or infiltrating inflammatory cells could have also confounded the gene expression profiles as observed. Gene expression profiles obtained from *in vitro* studies using airway and bronchial epithelial cells and from BALF cells might expand our understanding of cell specificity in O_3_ pulmonary toxicity, although the interactions of the various cell types might be lost.

The gene expression profiles derived in the present study provide insights into potential markers of the early O_3_ response. These markers must now to be evaluated at lower levels of O_3_ to establish a context within a dose–response model. The goal will be to use these profile maps to relate to mechanisms in human exposure scenarios.

## Figures and Tables

**Table 1:** Changes in BAL indicators in rats 2 hr after exposure to O_3_.^a^

Parameter	Air	2.0 ppm	5.0 ppm
Protein, μg/mL	96.5 ± 3.94	159.0 ± 8.91*	2,001.0 ± 348.0*
*N*-Acetylglucosaminidase	2.4 ± 0.28	3.82 ± 0.34*	18.0 ± 1.14*
Lysozyme, μg/mL	85.2 ± 1.71	79.5 ± 1.91*	71.4 ± 4.31*
Total cells, × 1,000/mL	37.2 ± 5.49	28.2 ± 2.36	30.9 ± 2.89
Neutrophils, %	0.60 ± 0.09	2.33 ± 0.87*	22.8 ± 4.47*
Ciliated cells, %	0.23 ± 0.16	6.07 ± 1.61*	40.4 ± 7.93*

a Results presented here are mean ± SE for six rats/group.

* Significantly different (*p* ≤ 0.05) by Student’s *t*-test.

**Table 2:** The number of differentially expressed (>2-fold) genes observed in rat lung tissue after 2-hr exposure to O_3_.^a^

Exposure concentration	No. of genes altered	Up-regulated	Down-regulated
2 ppm	Common	17	25
	Unique	9	11
	Total	26	36
5 ppm	Common	17	25
	Unique	9	6
	Total	26	31

a Results presented here show the number of genes that were altered (by ≥ 2-fold) and that were statistically significant by one-way ANOVA (*p* < 0.05). Genes that were common to both treatment groups and unique to each exposure group were derived by the Venn diagram approach in GeneSpring software, version 6.0, as detailed in ”Materials and Methods.”

**Table 3:** List of common genes induced (> 2-fold) in rat lung after 2-hr exposure to 2 and 5 ppm O_3_.^a^

Accession no.^b^	Gene symbol^c^	Gene name^c^	Fold change^d^
U72497	*Faah*	fatty acid amide hydrolase	14.17
M92848	*Ceacam1*	ecto-ATPase precursor (Cell-CAM105)	10.00
U17901	*Plaa*	phospholipase A-2 activating protein (PLAP)	7.96
U09793	*Kras2*	K-RAS 2B protooncogene	7.43
D14015	*Ccne1*	G1/S specific cyclin (cyclin E1)	5.57
L07736	*Cpt1a*	mitochondrial carnitine *O*-palmityltransferase	5.43
D10728	*Cd5*	T-cell surface glycoprotein (lymphocyte antigen CD5)	4.89
D44495	*Apex1*	apurinic/apyrimidinic endonuclease	4.86
X13722	*Ldlr*	low-density lipoprotein receptor	4.61
AF007789	*Plaur*	urokinase receptor	4.45
AF017437	*Cd47*	integrin-associated protein form 4	3.93
M91589	*Arrb1*	beta-arrestin 1	3.80
D10831	*Sell*	L-selectin precursor	3.50
X98490	*Rpa2*	replication protein A	3.38
M91590	*Arrb2*	beta-arrestin 2	2.41
L26267	*Nfkb1*	NF-kappa B transcription factor p105 subunit	2.38
X70871	*Ccng1*	G2/M specific cyclin G (cyclin G1)	2.11

a Genes that were induced and common to both 2- and 5-ppm–exposed rat lung are listed here.

b Accession numbers derived from the NCBI Unigene database (http://www.ncbi.nlm.nih.gov/).

c Gene symbols and names derived from the Duke Integrated Genomics Database (https://dig.cgt.duke.edu/try_query.php).

d Fold induction in gene expression. Fold changes in expression of these genes were statistically significant by one-way ANOVA (*p* < 0.05).

**Table 4:** List of common genes suppressed (> 2-fold) in rat lung after 2 hr exposure to 2 and 5 ppm O_3_.^a^

Accession no.^b^	Gene symbol^c^	Gene name^c^	Fold change^d^
U87306	*Unc5b*	transmembrane receptor UNC5H2	−33.3
J04486	*Igfbp2*	insulin like growth factor binding protein-2 (IGFBP-2)	−15.5 (2 ppm) −5.0 (5 ppm)
D26439	*Cd1d1*	rat CD1 antigen precursor	−10.78
M63334	*Cam4k*	calcium-calmodulin dependent protein kinase IV	−10.40
M31838	*Tacr2*	substance K receptor	−6.42
L27057	*Pde4a*	cAMP phosphodiesterase 4A	−5.14
V01217	*Actb*	cytoplasmic beta-actin	−4.58
X06890	*Rab4a*	ras-related protein RAB4A	−4.28
U87305	*Unc5a*	transmembrane receptor UNC5H1	−3.97
M64092	*Pkib*	PKI-beta cAMP protein kinase inhibitor	−3.73
M94056	*Dpep1*	dipeptidase	−3.64
L34067	*Gpc1*	glypican-1 precursor	−3.33
X13817	*Calm3*	calmodulin	−3.21
Z22867	*Pde3b*	cAMP-dependent phsophodiesterase	−3.21
AB004454	*Psen2*	presenilin2	−3.10
M59859	*Marcks*	miristoylated alanine-rich C-kinase substrate	−2.93
J05155	*Plcg2*	phospholipase C gamma 2	−2.93
J03754	*Atp2b2*	PMCA, calcium-transporting ATPase plasma membrane form	−2.92
X06889	*Rab3a*	ras-related protein RAB3A	−2.60
J03806	*Plcg1*	phospholipase C gamma 1	−2.57
U69278	*Epha3*	Eph-related receptor tyrosine kinase (Rek4)	−2.54
M32748	*Lif*	leukemia inhibitory/cholinergic neuronal differentiation factor	−2.44
M60525	*Vgf*	VGF nerve growth factor, inducible	−2.40
U34841	*Gprk5*	G-protein-coupled receptor kinase 5	−2.31
U06069	*Stxbp1*	Sec1; syntaxin binding protein 1	−2.11
M94043	*Rab38*	RAB-related GTP-binding protein	−2.02

a The genes that were found down-regulated/suppressed and common to both 2- and 5-ppm–exposed rat lung are listed here.

b Accession numbers derived from the NCBI Unigene database (http://www.ncbi.nlm.nih.gov/).

c Gene symbols derived from the Duke Integrated Genomics Database (https://dig.cgt.duke.edu/try_query.php).

d Fold suppression of gene expression. Fold changes in the expression of these genes were statistically significant by one-way ANOVA (*p* < 0.05).

**Table 5:** List of induced (> 2-fold) genes that are unique to 2 or 5 ppm O_3_.^a^

Accession no.*^b^*	Gene symbol^c^	Gene name^c^	Fold change^d^
2 ppm O_3_			
J03933	*Thrb*	thyroid hormone receptor beta, c-erbA-β	5.32
U73174	*Gsr*	glutathione reductase	5.21
L08447	*Cd3z*	T-cell receptor CD3 zeta subunit	4.37
L46791	*Ces3*	liver carboxylase precursor 10 (carboxylesterase 3)	3.95
J02650	*Rpl19*	60S ribosomal protein L19	3.51
X96394	*Abcc1*	multidrug resistance protein	2.70
D29766	*Bcar1*	FAK substrate p130	2.53
U49062	*Cd24*	signal transducer CD24	2.39
D16554	*Ubb*	polyubiquitin	2.25
5 ppm O_3_			
X17163	*Jun*	c-jun AP1	5.26
M84203	*Kcnc2*	potassium channel protein (KshIII A)	5.20
D10862	*Id1*	inhibitor of DNA binding 1	4.33
M81855	*Abcb1*	multidrug resistance protein 1	2.74
D14051	*Nos2*	inducible nitric oxide synthase	2.61
U45965	*Cxcl2*	Mip-2 chemokine ligand 2	2.57
M86389	*Hspb1*	heat shock 27 kDa protein 1	2.55
L29232	*Igf1r*	IGF-1 receptor	2.50
D16237	*Cdc25b*	M-phase inducer phosphatase 2	2.48

a Genes that were induced and unique to either 2- or 5-ppm–exposed rat lung are listed here. Accession numbers derived from the NCBI Unigene database (http://www.ncbi.nlm.nih.gov/).

c Gene symbols and names derived from the Duke Integrated Genomics Database (https://dig.cgt.duke.edu/try_query.php)

d Fold induction in gene expression. Fold changes in expression of these genes were statistically significant by one-way ANOVA (*p* < 0.05).

**Table 6:** List of suppressed (> 2-fold) genes that are unique to 2 or 5 ppm O_3_.^a^

Accession no.^b^	Gene symbol^c^	Gene name^c^	Fold change^d^
2 ppm O_3_			
J02999	*Rab2*	ras-related protein RAB2	3.50
L19698	*Rala*	GTP binding protein (Ral A)	3.11
X07287	*Pkrcg*	protein kinase C-γ	2.86
J03552	*Mug1*	plasma proteinase inhibitor	2.81
D85760	*Gna12*	guanine nucleotide-binding protein α-12	2.55
M99567	*Plcb3*	phospholipase C β-3	2.45
U00620	*Cfs2*	GM-CSF	2.45
M59980	*Kcnd2*	voltage-gated K+ channel protein	2.18
M83666	*Hck*	Hck tyrosine protein kinase, p56	2.15
AF020777	*Ptk2*	focal adhesion kinase	2.04
AF000300	*Lyn*	lyn A tyrosine kinase	2.03
5 ppm O_3_			
U46034	*Mmp11*	matrix metalloproteinase 11	3.61
D55627	*Rbl2*	retinoblastoma-like 2	3.49
M95738	*Slc6a11*	Na+/K+ dependent GABA transporter	2.95
M28647	*Atp1a1*	Na+/K+ ATPase α1 subunit	2.42
U93306	*Kdr*	VEGFR-2	2.16
M20637	*Plcd1*	phospholipase C delta 1	2.07

a The genes that are found suppressed/down-regulated and unique to either 2- or 5-ppm–exposed rat lung are listed here.

b Accession numbers from derived the NCBI Unigene database (http://www.ncbi.nlm.nih.gov/).

c Gene symbols and name s derived from the Duke Integrated Genomics Database (https://dig.cgt.duke.edu/try_query.php).

d Fold induction in gene expression. Fold changes in expression of these genes were statistically significant by one-way ANOVA (*p* < 0.05).

**Table 7:** Confirmation of gene array expression by real time RT-PCR for a select list of genes.^a^

		2 ppm		5 ppm	
Gene symbol^b^	Gene name^b^	Gene array	RT-PCR	Gene array	RT-PCR
*c-erb*	thyroid hormone receptor	5.0^c^	3.0	NC	NC
*c-jun*	transcription factor AP1	NC	NC	5.0	3.0
*Nos2*	inducible nitric oxide synthase	NC	NC	2.0	1.8
*Gsr*	glutathione reductase	5.0	5.2	NC	NC
*Igfbp2*	insulin-like growth factor binding protein 2	− < 15	−20.0	− < 5.0	−5.5

NC, no change in expression.

a Log numbers derived from real-time PCR analysis were normalized to the expression of the housekeeping gene GAPDH , which was unaltered by O_3_ exposure in rat lung tissue.

b Gene symbols and names derived from the Duke Integrated Genomics Database (https://dig.cgt.duke.edu/try_query.php).

c Fold change in expression compared with air-exposed control rat lung tissue.
